# A Geographic Information-Assisted Temporal Mixture Analysis for Addressing the Issue of Endmember Class and Endmember Spectra Variability

**DOI:** 10.3390/s17030624

**Published:** 2017-03-18

**Authors:** Wenliang Li, Changshan Wu

**Affiliations:** 1 Department of Geography, University of Wisconsin-Milwaukee, Milwaukee, WI 53201, USA; wenliang@uwm.edu; 2 School of Hydraulic Engineering, Changsha University of Science & Technology, Changsha 410114, Hunan, China

**Keywords:** logistic regression, classification tree, ordinary kriging, endmember class, endmember variability, temporal mixture analysis

## Abstract

Spectral mixture analysis (SMA) is a common approach for parameterizing biophysical fractions of urban environment and widely applied in many fields. For successful SMA, the selection of endmember class and corresponding spectra has been assumed as the most important step. Thanks to the spatial heterogeneity of natural and urban landscapes, the variability of endmember class and corresponding spectra has been widely considered as the profound error source in SMA. To address the challenging problems, we proposed a geographic information-assisted temporal mixture analysis (GATMA). Specifically, a logistic regression analysis was applied to analyze the relationship between land use/land covers and surrounding socio-economic factors, and a classification tree method was used to identify the present status of endmember classes throughout the whole study area. Furthermore, an ordinary kriging analysis was employed to generate a spatially varying endmember spectra at all pixels in the remote sensing image. As a consequence, a fully constrained temporal mixture analysis was conducted for examining the fractional land use land covers. Results show that the proposed GATMA achieved a promising accuracy with an RMSE of 6.81%, SE of 1.29% and MAE of 2.6%. In addition, comparative analysis result illustrates that a significant accuracy improvement has been found in the whole study area and both developed and less developed areas, and this demonstrates that the variability of endmember class and endmember spectra is essential for unmixing analysis.

## 1. Introduction

Spectral mixture analysis (SMA) is an inverse process that decomposes the spectra of a mixed pixel into areal fractions based on the pure spectra of its component land covers (also termed as endmembers) [1]. For successful SMA, the first step is the endmember selection, which involves the identification of types, numbers and corresponding spectra of endmembers [2,3]. Within this, some studies have been conducted for selecting the type and number of endmember classes, and several endmember class models have been proposed, such as vegetation-soil-shade (V-S-S) for estimating vegetation fraction in desert region [4], vegetation-impervious surface-soil (V-I-S) [5], vegetation-low albedo-high albedo (V-L-H) [6], and vegetation-low albedo-high albedo-soil (V-L-H-S) [7] for mapping urban biophysical composition. Although it is straightforward, only fixed endmember sets were adopted and applied for all pixels, which ignored the endmember class variability, and thereby making the estimation results less reliable. In order to address this issue, the first attempt was made by Roberts et al. [8]. With the consideration of endmember class variation, the multiple endmember spectral mixture analysis (MESMA) has been proposed and widely used. In addition, Zhang et al. [9] has proposed a prior-knowledge-based spectral mixture analysis, with which two different endmember class models, the V-L-H and V-L-H-S have been applied in high density areas and low density areas respectively. Zhang et al. [10] proposed an object-based unmixing analysis. In particular, the software of eCognition Developer was employed first to separate the image into several objects, and the unmixing analysis was conducted with endmember classes identified from each object. Furthermore, Li et al. [11] also developed a segmented and rule-based spectral mixture analysis for estimating imperviousness. Specifically, the study area was divided into segmented regions, and different endmember class sets were selected for unmixing analysis in each region. Recently, Li and Wu [12] analyzed the endmember class variation with the help of spatial information, and satisfactory estimation results were achieved for large-scale impervious surfaces estimation.

In addition to the aforementioned endmember class selection, the endmember spectra extraction is the other important step for implementing SMA. Traditionally, the endmember spectra are extracted from the vertices of an n-dimensional scatter plot manually or automatically (e.g., N-FINDR [13], Pixel Purity Index (PPI) [14]). As a consequence, a fix set of endmember spectra was applied for the whole image for unmixing analysis. As in fact of the complexity of natural and urban landscapes, the endmember spectra variability may have not been fully considered, which in turn result in significant estimation error [8,15,16]. Therefore, endmember spectra variability has been considered as a profound error source for SMA. Endmember spectra variability can be grouped into two categories: within-class and between-class variability. The within-class variability refers to the spectral variations of the same land use/cover, such as the tiles, concrete, asphalt of imperviousness. The between-class variability refers to the spectral confusion between various land uses/covers, such as the confusion of bare soil and impervious surfaces. In order to address the challenging issue, numerous models have been developed, including AutoSWIR [15], derivative spectral unmixing (DSU) [17], Normalized Spectral Mixture Analysis [18], etc. In addition, spectral weighting techniques (weighted spectral mixture analysis, WSMA) [19] and two-step weighting SMA [20]), continuous wavelets techniques [21], spectral modeling approaches [22], iterative mixture analysis (MESMA) [8], neighbourhood-specific endmember signature generation technique [23] and geostatistical technique (Geostatistical temporal mixture analysis, GTMA [24]) have also been proposed for addressing the endmember spectra variability issue.

While numerous approaches have been developed and widely used for addressing the endmember class and endmember spectra variability issue, some challenging problems still remain. Specifically, in most studies, endmember classes are still selected based on the n-dimensional feature spaces and finally a fixed set of endmember classes and averaged pure endmember spectra are applied in each pixel of the whole image. However, the physical meaning of endmember classes and local variation of endmember spectra has been totally neglected. In fact, on the one hand, the spatial distribution of endmember classes is majorly associated with global environmental and socio-economic driving forces such as elevation, slope, distance to water, distance to city, etc. For instance, urban areas tend to be distributed along the areas with gentle slope and convenient access to transportation networks, and vegetated areas are mainly located around lakes, river streams with gentle elevation. On the other hand, due to the spatial variations of natural and social environment, the endmember spectra tend to vary within a study area. For example, the spectra of impervious surfaces are varied spatially due to the heterogeneity of the materials (low albedo: asphalt, high albedo: glass and plastic), the spectra of vegetation might vary depending on the chlorophyll content and the shape, angle and density of the canopy elements. Therefore, instead of using one set of endmember classes and spectra, the spatially varied endmember classes and localized endmember spectra become essential for unmixing analysis. In addition, until now, most approaches only focussed on addressing either the issue of endmember class variability or endmember spectra variability; few studies have been conducted for both of them. In fact, the selection of endmember class is the foundation of endmember spectra extraction, and they are all serving as the key to successfully SMA. Therefore, both the issue of endmember class variability and endmember spectra variability needs to be seriously considered.

To address the aforementioned challenging problems, in this study, we proposed a geographic information-assisted temporal mixture analysis (GATMA). In particular, we utilized logistic regression analysis to analyze the relationship between land use/land covers and surrounding environmental and socio-economic forces (e.g., elevation, slope, and distance to the nearest city, town, river, lake, etc.) and derive the global spatial distribution probability of land use/land covers. Furthermore, a classification tree method was applied to identify the existence of endmember classes at each pixel in the whole study area. With the quantified per-pixel endmember classes, an ordinary kriging analysis was conducted to generate the ‘purest’ endmember spectra for each pixel. The proposed model can be detailed as three steps: (1) identifying the numbers and types of endmember classes for each pixel through logistic regression analysis and classification tree methods; (2) extracting spatially varied endmember spectra using ordinary kriging analysis; and (3) estimating land use/land cover fractions using temporal mixture analysis. The rest of the article is organized as follows. The study area and data are described in Section 2; the proposed GATMA is introduced in Section 3; estimation results of the proposed GATMA, accuracy assessment, and comparative analysis with the Phenology-based Temporal Mixture Analysis (PTMA) and Phenology-based Multiple Endmember Temporal Mixture Analysis (PMETMA) are listed in Section 4; and finally, discussion, conclusions and future research directions are provided in Section 5 and Section 6.

## 2. Study Area and Data

**Figure 1:**
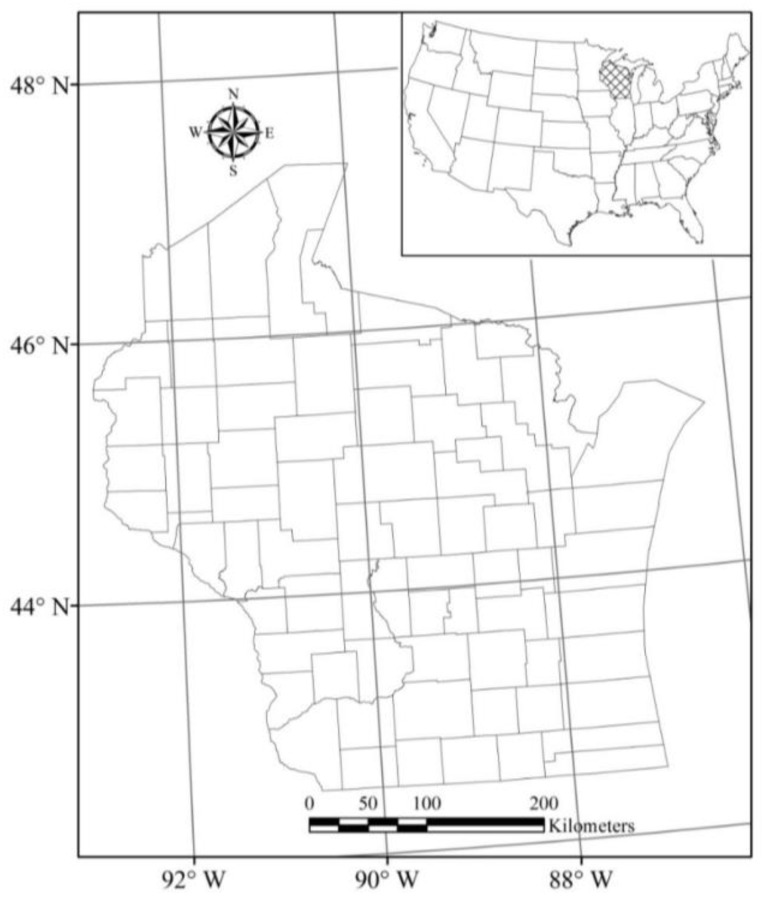
The study area: Wisconsin, USA.

The selected study area, Wisconsin State, is located in the Midwest of the United States, and is composed of 72 counties with a geographic area of 169,639 km^2^ (see Figure 1). Wisconsin comprises a large number of natural landscapes, including deciduous and evergreen forest in the north, agriculture lands in the southwest, and pasture in the northwest. In contrast, most urbanized areas are located in the south-eastern Wisconsin, including the capital city, Madison and the state’s most populated area of Milwaukee. According to the data reported by United States Census Bureau on July 2013, the total population in Wisconsin was 5.74 million, increased about 16% in the past two decades, and approximately 68% of the population are living in urban areas and 35% of the population resides in the Southeastern Wisconsin. While the rapid population growth and urbanization brings some economic benefits, it also raises several environmental issues, including urban heat island effect, biodiversity reduction, etc. Therefore, accurate estimations of urban growth and impervious surface fractions have become essential in the study area.

In order to carry out the proposed GATMA for deriving the impervious surface fractions in the state of Wisconsin, the Moderate-resolution Imaging Spectroradiometer (MODIS) NDVI data in 2006 were collected and utilized in this study. In particular, the product of MOD13Q1 was collected every half month from the United States Geological Survey (USGS) website (www.usgs.gov) with a positional accuracy of 0.5 pixels and spatial resolution of 250 m respectively. Moreover, due to large cloud cover and bad weather condition, one image in early November was removed and finally 22 MODIS NDVI images was obtained and applied for spectral unmixing. Furthermore, the National Land Cover Data (NLCD) 2006 was collected for identifying land use/land cover types and the NLCD 2006 Percent Developed Imperviousness data was obtained for evaluating the performances of the proposed GATMA (all collected from Multi-Resolution Land Characteristics Consortium (MRLC), www.mrlc.gov/nlcd06_data.php). In addition, the city, village distribution data, and transportation data (e.g., road, railway) were collected from the American Geographical Society Library at the University of Wisconsin-Milwaukee. Moreover, the hydrological data (e.g., river, lake) and elevation data were obtained from the USGS website, and the slope data was generated using ArcGIS 10, a commercial GIS package.

## 3. Methodology

**Figure 2:**
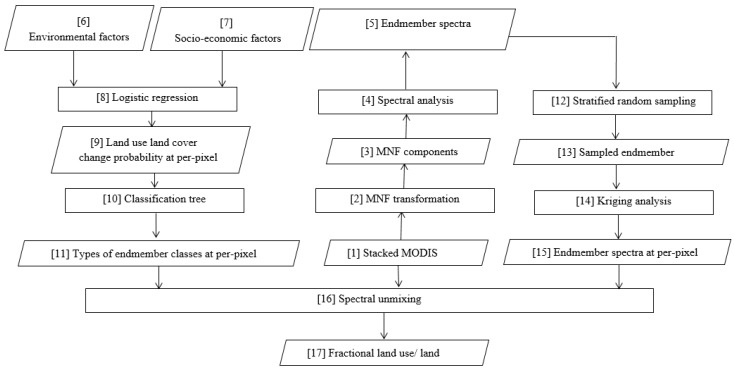
Flowchart of the proposed geographic information-assisted temporal mixture analysis (GATMA) approach.

**Figure 3:**
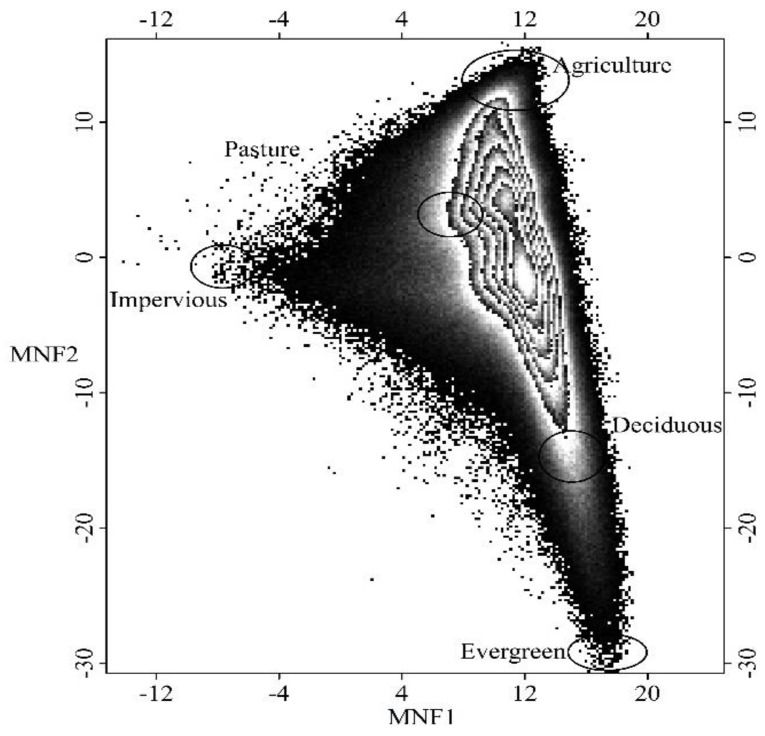
2-dimensional scatter plot generated from minimum noise fraction (MNF) components.

To carry out the proposed GATMA approach, three major steps were conducted, including the identification of the numbers and types of endmember classes, the extraction of spatially varied endmember spectra, and the estimation of fractional land use/land covers. Before conducting the proposed method, we identified potential endmembers and corresponding pure spectra using the minimum noise fraction (MNF) transformation (Step 2 in Figure 2) based on the stacked temporal sequences of MODIS data (Step 1 in Figure 2). In particular, ENVI—a commercial remote sensing program—was used for conducting MNF transformation. With the MNF transformation, approximately 90% of the variances were preserved in the first three components, and spectral correlations among spectral bands were effectively removed. Therefore, we generated several feature spaces using the first three MNF components (Step 3 in Figure 2), and endmember pure spectra (Step 5 in Figure 2) were extracted from the vertices of the feature space scatterplots (see Figure 3) through spectral analysis (Step 4 in Figure 2). Finally, five endmember classes (including agriculture, impervious surface, deciduous forest, evergreen forest, and pasture) and corresponding pure spectra were identified and extracted from the scatterplots of the first three MNF components and further verified with the observed data.

### 3.1. The Identification of Endmember Class Type and Number

In order to automatically identify the type and number of endmember classes for each pixel throughout the whole study area, the logistic regression analysis approach and classification tree method were applied. In particular, a logistic regression analysis (Step 8 in Figure 2) was utilized to derive spatial distribution probability of endmember classes (Step 9 in Figure 2) through analyzing the spatial relationship between land use/land covers and their surrounding environmental (Step 6 in Figure 2) and socio-economic forces (Step 7 in Figure 2), and the classification tree method (Step 10 in Figure 2) was employed to identify the existence status (present or absent) (Step 11 in Figure 2) of endmember classes for each individual pixel. In this study, five land use/land covers (agricultural land, built-up, deciduous forest, pasture, and evergreen forest) and eight driving forces (DEM, slope, the distance to the nearest road, railway, city, village, river, and lake) were considered in the logistic regression model, and the spatial distribution probability of endmember classes were derived by the following formula,

(1) $$Log\left(\frac{P_{i,j}}{1-P_{i,j}}\right)=\alpha_{0}+\alpha_{1}X_{1,i}+\alpha_{2}X_{2,i}+\cdots +\alpha_{n}X_{n,i}$$

where *P_i,j_* is the distribution probability of endmember class *i* at pixel *j*; *X_n,i_* is the driving forces *j* for endmember class *i*, and *α_n_* is the coefficient for the driving forces *n*. In this study, 2000 samples of each endmember class were collected based on the NLCD 2006 data for constructing the logistic regression analysis model.

With the help of the derived spatial distribution probability of land use/land covers, the identification of numbers and types of endmember classes were implemented through classification tree method. Specifically, 1000 samples were selected (200 samples for each endmember class were randomly selected from the spatial distribution map of five endmember classes within the study area based on NLCD 2006 data) and input into the program of SEE5 to analyze the relationship between endmember class status (categorical variable) and the spatial distribution probability of endmember classes (independent variables) for each individual pixel. SEE5, also termed as C5.0, is a widely used commercial program developed by Quinlan (1996) for generated decision trees [25] (https:www.rulequest.com/see5-info.html). After implementing the classification tree method, we successfully generated rules to identify the numbers of endmember classes and the specific endmember class types in each pixel.

### 3.2. The Extraction of Spatial Varied Endmember Spectra

With the identified endmember classes at each pixel, the next step is to extract pure endmember spectra of each endmember class at the per-pixel level. We obtained pure represented endmember spectra (Step 5 in Figure 2) from the vertices of the 2-D scatterplot through spectra analysis (Step 4 in Figure 2). Then a stratified sampling scheme (Step 12 in Figure 2) was applied to collect 200 endmember samples (Step 13 in Figure 2) for agriculture, deciduous forest, evergreen forest, and pasture classes, and only 50 samples were collected for impervious surfaces due to the lack of pure pixels. Furthermore, a spatially varied endmember spectra extraction was conducted for quantifying per-pixel level ‘pure’ endmember spectra of each endmember class (90% of endmember samples were used for interpolation, 10% endmember samples were reserved for further evaluating interpolation accuracy). In this study, ‘pure’ endmember spectra (Step 15 in Figure 2) were derived through applying the ordinary kriging analysis (Step 14 in Figure 2). Ordinary kriging is a geo-statistical approach and commonly viewed as an interpolation technique, and has been widely applied in many fields. The implementation of ordinary kriging is based on the lease square optimization principle and can be conducted through implementing three major steps: (1) variogram estimation for spatial structure analysis; (2) model fitting; and (3) weight estimation and interpolation.

In order to analyze the spatial structure of all sample points, the experimental variogram can be calculated in the first step by the following formulation:

(2) $$\hat{\gamma }\left(d\right)=\frac{1}{2\left(n\right)}\sum_{j=1}^{n}{\left[NDVI_{i,a}\left(x_{j}\right)-NDVI_{i,a}(x_{j}+d)\right]}^{2}$$

where, $\hat{\gamma }\left(d\right)$ is the variogram, *NDVI_i,a_* (*x_j_*) and *NDVI_i,a_* (*x_j_* + *d*) are known ‘pure’ NDVI values of endmember *i* in band *a* at locations *x_j_* and *x_j_* + *d*, respectively, *d* is a lag to describe the distance and direction between location *x_j_* and *x_j_* + *d*, and *n* is the total number of sample point pairs separated by the vector *d*.

With the knowledge of the spatial structure extracted from the experimental variogram calculation, a fitted theoretical model need to be selected. The theoretical variogram model is classified according to the function type, and for more discussions can be found in Curran and Atkinson (1998) [26] and McBratney and Webster (1986) [27].

Finally, the ‘pure’ *NDVI* value for endmember *i* in band *a* at pixel *k* can be estimated by

(3) $$NDVI_{i,a,k}=\sum_{j=1}^{n}W_{j}NDVI_{i,a,j}$$

where *NDVI_i,a,k_* is the estimated ‘pure’ *NDVI* value of endmember *i* in band *a* at pixel *k*, *NDVI_i,a,j_* is the known ‘pure’ *NDVI* values of endmember *i* in band *a* at location *j*, and *W_j_* are the weights that should be calculated.

Subject to

(4) $$\sum_{j=1}^{n}W_{j}=1$$

In this study, the experimental variograms were conducted using GS + 7.0 (Geostatistics for the Environmental Sciences) to find the best fit theoretical model, and ArcGIS10 was used to implement ordinary kriging for interpolating localized endmember spectra.

### 3.3. Temporal Mixture Analysis

With the extracted pixel level adaptive endmember classes and corresponding spectral signature, a linear temporal mixture analysis (Step 16 in Figure 2) was implemented for the estimation of land use/land cover fractions (Step 17 in Figure 2). Temporal mixture analysis is an inverse model that a mixed *NDVI* spectral signature is decomposed into areal abundances of its pure land cover components (also termed as endmember classes) through least squares optimization model. The temporal mixture analysis can be conducted with non-constrains and full-constrains, in this study, two constrains including the fractions of all endmember classes non-negative and sum to one were applied to make the unmixing results to be presented with physical meanings. The temporal mixture analysis and two constrains can be formulated as follows:

(5) $$NDVI_{b}=\sum_{i=1}^{N}f_{i}\times NDVI_{i,b,m}+e_{b}$$

Subject to $\sum_{i=1}^{N}f_{i}=1$ and $f_{i}\geq 0$

where *NDVI_b_* is the mixed *NDVI* spectral signature for band *b*, *N* is the total number of all identified endmember classes, *f_i_* is the Abundances of endmember *i*, *NDVI_i,b,m_* is the ‘pure’ *NDVI* spectral signature of endmember *i* in band *b* at pixel *m*, and *e_b_* is the residual. In order to assess the model fitness, the *e_b_* and *RMS* were applied.

(6) RMS=(∑b=1Meb2M)12

where *M* is the number of bands in the remote sensing image.

### 3.4. Comparative Analysis and Accuracy Assessment

For comparative purposes, we have also implemented PTMA and PMETMA. With PTMA, only one endmember set (generated through averaging all pure endmembers) was employed to quantify the fraction of land use land covers for the whole study area through fully constrained TMA. For PMETMA, the issue of endmember class and spectra variability has been considered, it allows the variety of endmember set, and the best fit model are selected for unmixing analysis. For detailed information about PTMA and PMETMA, readers can refer to [28].

In order to evaluate the performance of the proposed geographic information-assisted TMA approach, the NLCD 2006 Percent Developed Imperviousness data was collected and applied as the reference data, three widely applied measures including root mean square error (*RMSE*, Equation (7)), systematic error (*SE*, Equation (8)) and mean absolute error (*MAE*, Equation (9)) were calculated to evaluate the estimation accuracy of the proposed TMA approach. The *SE* and *MAE* can be calculated as follows:

(7) $$RMSE=\sqrt{\frac{\sum_{i=1}^{N}{\left(\bar{A_{i}}-A_{i}\right)}^{2}}{N}}$$

(8) $$SE=\frac{\sum_{i=1}^{N}\left(\bar{A_{i}}-A_{i}\right)}{N}$$

(9) $$MAE=\frac{\sum_{i=1}^{N}\left|A_{i}-\bar{A_{i}}\right|}{N}$$

where $\bar{A_{i}}$ is the modelled impervious surface abundance from the proposed TMA approach for pixel *i*, *A_i_* is the obtained NLCD 2006 impervious surfaces abundances for pixel *i*, and *N* is the total number of pixels.

## 4. Results

### 4.1. Spatially Varied Endmember Class Identification

The logistic regression analysis has been successfully implemented and then the spatial distribution probability of all endmember classes were generated. Table 1 shows the spatial relationship between endmembers and surrounding environmental and socio-economic factors. Taking the built-up class as an example, it only positively associated with the slope and distances to the nearest railway, and negatively associated with all other driving factors. In order to assess the fitness of the regression model, the relative operating characteristic (ROC) was calculated and the ROC values for all models are over 0.65, indicating that the all chosen driving factors can be used to explain the spatial distribution of all land use types.

With the knowledge of the spatial distribution probabilities of all endmember classes produced from the logistic regression model, the classification tree approach was utilized to automatically identify the present and absent of endmember classes for each pixel (Table 2). For instance, the built-up endmember is present only within three conditions: (1) the distribution probability of built up is over 9.3%; (2) the distribution probability of built up is smaller than 9.3% but the probability of evergreen is over 0.6%; and (3) the probability of built up is lower than 7.8% coupled with the probability of agriculture is over 44.1%. They are corresponding with three typical land use patterns respectively which are (1) urbanized areas, (2) urbanized areas with forest, and (3) urbanized area with agricultural land.

### 4.2. Spatially Varied Endmember Spectra Extraction

**Figure 4:**
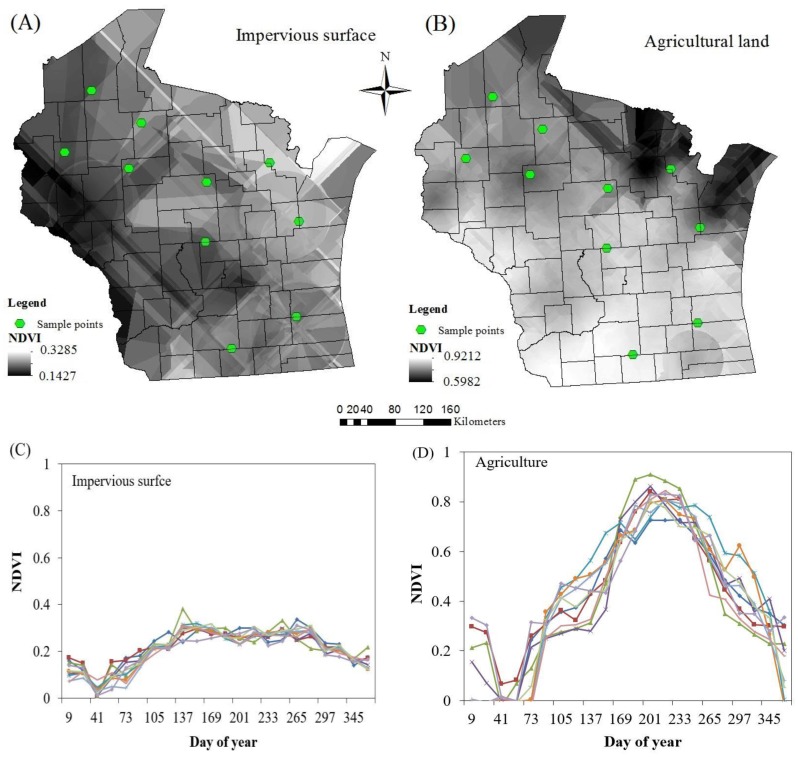
Localized endmember spectra using ordinary kriging: (**A**) Impervious surface, (**B**) agriculture in late July and sampled spectral signature, (**C**) impervious surface, (**D**) agriculture.

In order to extract spatially varying endmember spectra for each endmember class, the ordinary kriging method was applied for (1) estimating experimental variogram, (2) fitting a mathematical function, and (3) interpolating endmember using the weights derived from the function. In particular, the variogram of endmember classes were analyzed based on the collected ‘pure’ samples. Further, several models were applied to fit the variograms and the best fit model was selected for interpolating the per-pixel endmember spectra for all endmember classes, including impervious surfaces, pasture, evergreen forest, agricultural lands, and deciduous forest. In order to evaluate the interpolated endmember spectra, 10% of collected endmember samples were reserved. The mean absolute error (MAE) was calculated through comparing the reference endmember samples and estimated endmember spectra. Comparison results show that the MAEs of impervious surface, agricultural land, deciduous forest, evergreen forest, and pasture are 3.43%, 3.15%, 2.29%, 5.53%, and 5.49% respectively, and the estimated endmember spectra is accurate enough for further study. Taking agriculture and impervious surfaces as examples, Figure 4 indicates the spatial variation of the estimated endmember spectra. Taking the NDVI value in late July (NDVI band 13) of agriculture endmember as an example, its value decreases from southwest to northeast in the study area, with the NDVI value changing from 0.9212 to 0.5982. For impervious surfaces, the NDVI value decreases from northern Wisconsin to the southern portion with the highest value at 0.3285 and lowest value at 0.1427. In order to further prove the existence of spatial variation, we also randomly selected 10 sample points and extracted corresponding endmember spectra for agriculture and impervious surfaces, generating the charts in Figure 4C,D.

### 4.3. Fully Constrained Temporal Mixture Analysis

**Figure 5:**
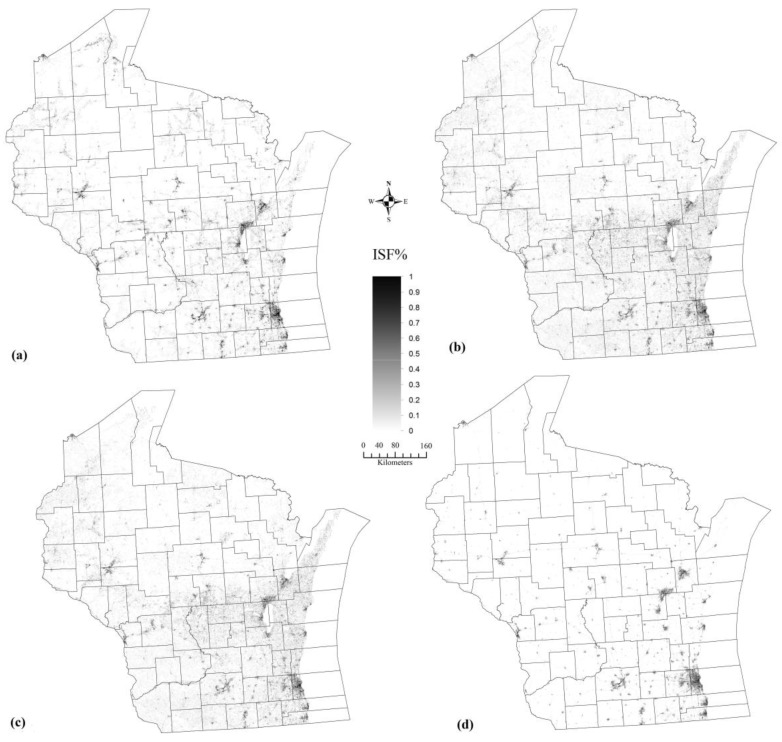
Impervious surfaces fraction maps generated from (**a**) GATMA method (**b**) Phenology-based Multiple Endmember Temporal Mixture Analysis (PMETMA) method (**c**) Phenology-based Temporal Mixture Analysis (PTMA) method and the reference map (**d**) ISF2006.

With the identified spatially varying endmember classes and spatially varying pure endmember spectra (see Figure 4), a fully constrained linear temporal mixture analysis was implemented. The modelled impervious surface fraction image (see Figure 5a) illustrates that it highly correlates with the actually distribution in the study area. In particular, the high fractional impervious surfaces (70%–100%) are distributed majorly in urbanized areas such as Milwaukee, Madison and Green Bay, medium fractional impervious surfaces (30%–70%) are mainly located at suburban areas, such as Racine, Mequon, Kenosha, and Grafton, around the above mentioned three big cities. Low fractional impervious surfaces (0%–30%) could be easy recognized in northern and western parts of Wisconsin which is majorly covered by forest and agricultural lands. A quantitative analysis has also been conducted and the results are reported in Table 3. Results show that the proposed GATMA has achieved a promising performance for the impervious surfaces estimation in Wisconsin with an RMSE of 6.81%, SE of 1.29% and MAE of 2.6%. In particular, a much better performance has been achieved for less developed areas with the RMSE of 6.43%, SE of 1.41% and MAE of 2.42% compared with the developed areas with the RMSE of 16.77%, SE of −3.96% and MAE of 10.99%.

### 4.4. Comparative Analysis

For the comparative purpose, we also implemented the PTMA and PMETMA and the results are included in Figure 5. Results show that the spatial distribution pattern of the modelled maps from the proposed GATMA, PTMA and PMETMA are very similar. That is, high fractional impervious surfaces are distributed in the southeast Wisconsin and low fractional impervious surfaces are located in the northern and western Wisconsin. In addition, quantitative analysis result shows that a much worse performance has been discerned using both PTMA and PMETMA, with an RMSE of 7.27% and 7.54%, SE of 3.25% and 2.13%, MAE of 4.03% and 3.36%. Detailed analysis shows that a much significant underestimation has been derived for developed areas by PTMA and PMETMA with the RMSE of 15.41% and 15.62%, SE of −8.17% and −7.46%, MAE of 12.15% and 12.16% respectively. For less developed areas, the performances of PTMA and PMETMA are also worse than the proposed GATMA approach, as a much higher overestimation can be detected with the RMSE of 7.07% and 7.51%, SE of 3.43% and 2.32%, MAE of 3.9% and 3.23%. In summary, compared with the PTMA and PMETMA, a better performance has been achieved by the proposed GATMA for the whole study area, and also for both developed and less developed areas.

## 5. Discussion

The selection of the endmember class and endmember spectra has been widely assumed as key to successful SMA. As a result of the spatial heterogeneous nature of landscapes, the distribution of endmember classes and corresponding spectra tends to illustrate spatial variability. Until now, however, the examined endmember classes and extracted endmember spectra are usually considered as identical throughout the whole study area. While MESMA assumes the endmember class variations, the endmember class has been selected only based on the per-pixel error indices such as RMSE, EAR, MASA, and IES without considering any geographic information. Therefore, the issue of endmember class and endmember spectra variability has been somewhat ignored and may lead to less satisfied land use fraction estimation. With the proposed GATMA, the aforementioned issues could be successfully addressed. Specifically, a logistic regression analysis was applied to quantify the global land use distribution probability information and further a classification tree method was utilized to analyze the extracted probability information to identify the present status of each endmember class throughout the whole study area. Moreover, an ordinary kriging was employed to extract a spatially varied endmember spectra at all pixels in the remote sensing image. Consequently, the globally selected endmember classes with localized endmember spectra information can greatly improve the performance of the unmixing analysis. The research results also indicate that it is necessary to consider the issue of endmember class and endmember spectra variability, and a much better estimation accuracy has been achieved for the whole study area and both the developed and less developed areas.

## 6. Conclusions and Future Research Directions

In this study, we developed a geographic information-assisted temporal mixture analysis (GATMA) for modelling the impervious surface distribution in the state of Wisconsin. In particular, a logistic regression analysis was applied for examining the relationship between endmember classes and surrounding environmental and social-economic factors; furthermore, a classification tree method was utilized to quantify the spatially varied endmember class distributions for addressing the issue of endmember class variability. Moreover, an ordinary kriging analysis was employed to generate spatially varied endmember spectra for each endmember class to overcome the issue of endmember spectra spatial variability. Finally, the fully constrained linear temporal mixture analysis was conducted to generate the impervious surface distributions.

Analysis of results shows that the developed GATMA has achieved a promising accuracy for estimating the fraction of impervious surfaces with the RMSE of 6.81%, SE of 1.29% and MAE of 2.6% for the whole study area. Further analysis indicates that a satisfactory performance has also been achieved for both developed area and less developed area with slightly underestimation and slightly overestimation respectively. In order to further evaluate the proposed model performance, a comparison study has also been conducted between the developed GATMA and the PTMA, PMETMA. Comparative results suggest that the proposed GATMA achieved a promising performance. In particular, compared with the PTMA and PMETMA, the RMSE value for the whole study area decreased 6.33%% and 9.68%, the SE value decreased 60.31% and 39.44%, and the MAE decreased 35.48% and 22.62% respectively. Furthermore, for the developed area, while the RMSE value slightly increased 8.83% and 7.36%, the SE value significantly decreased 51.53% and 42.96%, and the MAE value decreased 9.55% and 9.62% respectively. For the less developed area, the RMSE value decreased 9.05% and 14.38%, the SE value decreased 58.89% and 39.22%, and the MAE value decreased 37.95% and 25.08% respectively. In summary, the proposed GATMA achieved a better performance than the PTMA and PMETMA as it has successfully addressed the endmember class and endmember spectra variability issue in unmixing analysis.

Although the proposed GATMA has achieved a promising accuracy for estimating impervious surfaces fractions, its ability in separating impervious surface from other land uses/land covers is still unclear, mainly due to the unavailability of other land use observation data at a large-scale. Therefore, one future research direction could be the collection of other land use reference data for further validation of the proposed GATMA approach. Furthermore, in this study, only MODIS NDVI data has been used, and it is necessary to evaluate the performance of the proposed GAMTA approach when applied to other dataset (e.g., hyperspectral image, Landsat TM, SPOT, etc.).

## Figures and Tables

**Table 1:** The logistic regression results for land uses/covers.

Driving Forces	Deciduous Forest	Evergreen Forest	Agricultural Land	Pasture	Built-Up
Constant	−4.0493307	−3.7247195	−0.326205	−1.7224049	−0.67114
Slope (degree)	0.3214467	−0.2221291	−0.1495531	−0.0361478	0.0020756
Elevation (m)	0.0081379	−0.0010718	0.0006489	−0.0001815	−0.0023155
Distance to road (m)	8.85 × 10^−6^	2.47 × 10^−6^	−0.0001429	−0.0001007	−0.0002108
Distance to railway (m)	−1.52 × 10^−6^	−1.959 × 10^−5^	8.08 × 10^−6^	−6.94 × 10^−6^	1.45 × 10^−6^
Distance to city (m)	0.0000263	1.151 × 10^−5^	−2.835 × 10^−5^	−3.098 × 10^−5^	−2.211 × 10^−5^
Distance to village (m)	−1.629 × 10^−5^	1.257 × 10^−5^	−6.065 × 10^−5^	−3.284 × 10^−5^	−9.88 × 10^−5^
Distance to river (m)	−3.618 × 10^−5^	−8.011 × 10^−5^	1.284 × 10^−5^	3.977 × 10^−5^	−3.483 × 10^−5^
Distance to lake (m)	3.733 × 10^−5^	0.0001365	0.0001318	0.0001185	−5.401 × 10^−5^
ROC	0.756	0.667	0.761	0.726	0.747

**Table 2:** Endmember class identification rules from classification tree.

		Deciduous Forest	Evergreen Forest	Agricultural Land	Pasture	Built-Up
Deciduous	Present					>0.169426
forest		>0.453019				≤0.169426
				>0.388859		>0.064593
	Absent	≤0.453019		≤0.388856		≤0.169426
		≤0.453019		>0.388856		≤0.064593
Evergreen	Present	>0.203029	>0.021945		0.111230–0.114748	
forest		>0.203029	<0.021945			
	Absent	≤0.203029				
		>0.203029	≤0.021945		>0.114748	
		>0.203029	≤0.021945		<0.111230	
Agricultural	Present		<0.052188	>0.144238		
land				<0.144238		0.110385–0.244997
	Absent		≥0.052188	≥0.144238		
				≤0.144238		≥0.244997
				≤0.144238		≤0.110385
Pasture	Present			>0.144533	>0.116225	
	Absent			≤0.144533		
				>0.144533	<0.116225	
Built-up	Present			>0.441149		≤0.077754
						>0.093196
			>0.005930			≤0.093196
	Absent			≤0.441149		≤0.077754
			≤0.005930			≤0.093196

**Table 3:** Accuracy assessment of impervious surfaces with the proposed GATMA, PTMA and PMETMA.

		GATMA (%)	PTMA (%)	Percent Difference (%)	PMETMA (%)	Percent Difference (%)
Overall	RMSE	6.81	7.27	−6.33	7.54	−9.68
	SE	1.29	3.25	−60.31	2.13	−39.44
	MAE	2.6	4.03	−35.48	3.36	−22.62
Developed	RMSE	16.77	15.41	8.83	15.62	7.36
	SE	−3.96	−8.17	−51.53	−7.46	−42.96
	MAE	10.99	12.15	−9.55	12.16	−9.62
Less	RMSE	6.43	7.07	−9.05	7.51	−14.38
developed	SE	1.41	3.43	−58.89	2.32	−39.22
	MAE	2.42	3.90	−37.95	3.23	−25.08
